# Activation of MET pathway predicts poor outcome to cetuximab in patients with recurrent or metastatic head and neck cancer

**DOI:** 10.1186/s12967-015-0633-7

**Published:** 2015-08-29

**Authors:** Juan Madoz-Gúrpide, Sandra Zazo, Cristina Chamizo, Victoria Casado, Cristina Caramés, Eduardo Gavín, Ion Cristóbal, Jesús García-Foncillas, Federico Rojo

**Affiliations:** Molecular Pathology Laboratory, IIS-Fundacion Jimenez Diaz, UAM, Avda. Reyes Catolicos 2, 28040 Madrid, Spain; Oncology Department, Fundacion Jimenez Diaz, Madrid, Spain; Pathology Department, IIS-Fundacion Jimenez Diaz, UAM, Avda. Reyes Catolicos 2, 28040 Madrid, Spain; Translational Oncology Department, IIS-Fundacion Jimenez Diaz, UAM, Madrid, Spain

**Keywords:** Head and neck squamous cell carcinoma (HNSCC), EGFR, MET, HGF, Cetuximab, Prognostic factor

## Abstract

**Background:**

Activation of the *MET* oncogene promotes tumor growth, invasion and metastasis in several tumor types. Additionally, MET is activated as a compensatory pathway in the presence of EGFR blockade, thus resulting in a mechanism of resistance to EGFR inhibitors.

**Methods:**

We have investigated the impact of HGF and MET expression, MET activation (phosphorylation), *MET* gene status, and *MET*-activating mutations on cetuximab sensitivity in recurrent or metastatic squamous cell carcinoma of the head and neck (HNSCC) patients.

**Results:**

A single-institution retrospective analysis was performed in 57 patients. MET overexpression was detected in 58 % patients, *MET* amplification in 39 % and MET activation (p-MET) in 30 %. Amplification was associated with MET overexpression. Log-rank testing showed significantly worse outcomes in recurrent/metastatic, MET overexpressing patients for progression-free survival and overall survival. Activation of MET was correlated with worse PFS and OS. In multivariate logistic regression analysis, p-MET was an independent prognostic factor for PFS. HGF overexpression was observed in 58 % patients and was associated with MET phosphorylation, suggesting a paracrine activation of the receptor.

**Conclusions:**

HGF/MET pathway activation correlated with worse outcome in recurrent/metastatic HNSCC patients. When treated with a cetuximab-based regimen, these patients correlated with worse outcome. This supports a dual blocking strategy of HGF/MET and EGFR pathways for the treatment of patients with recurrent/metastatic HNSCC.

**Electronic supplementary material:**

The online version of this article (doi:10.1186/s12967-015-0633-7) contains supplementary material, which is available to authorized users.

## Background

The incidence of head and neck cancer is increasing worldwide, and has recently become the sixth most common malignancy [1]. Malignancies of the head and neck are associated with tobacco use, alcohol consumption, and Epstein–Barr virus (EBV) and/or papillomavirus (HPV) infections [2]. Advances in diagnosis, prevention, and management of advanced cases have been made in recent years, and while long-term survival rates have improved [1], they remain some of the lowest among major cancer types worldwide. Head and neck squamous cell carcinoma (HNSCC) is a particularly prevalent type of head and neck cancer, constituting 90 % of all head and neck cancers. Survival rates are low due to late diagnosis at advanced stages, the failure of treatment [3], and the development of secondary malignant tumors. These problems underscore the importance of improving strategies of primary chemotherapy and chemoprevention of HNSCC.

Traditionally, the concurrent use of surgery, radiation, and/or multiagent chemotherapy for the management of patients with late-stage, locoregionally advanced unresectable disease has been the standard for treatment of HNSCC [4]. Alternatively, focus has shifted in recent years towards biological therapies [5]. These include drugs that target growth factors and their receptors, signal transduction, cell cycle control, protein degradation, hypoxia, and angiogenesis. Epidermal growth factor receptor (EGFR) is a receptor tyrosine-kinase that is overexpressed in 90 % of HNSCC tumors and is involved in tumor growth, invasion, metastasis, and angiogenesis [6]. It is an early marker of carcinogenesis in HNSCC, and has been associated with a poor outcome [7]. This makes it a reasonable target for specific biological drugs [8], from antibodies to small-molecule inhibitors. Cetuximab is a chimeric IgG1 monoclonal antibody that inhibits ligand binding to the EGFR extracellular domain [9] (hence interfering with receptor activation) and enhances the activity of chemotherapeutic agents [10]. Numerous clinical trials with cetuximab have improved the treatment of recurrent/metastatic HNSCC both as first-line therapy and following failure of platinum-based chemotherapy [11, 12]. Since its approval for HNSCC in 2006, the clinical data produced suggest that cetuximab plays an important role in the locoregional treatment of these pathologies [13]. A second anti-EGFR strategy targets the intracellular domain of the receptor with low-molecular-weight tyrosine kinase inhibitors (erlotinib, gefitinib) and influences downstream signaling processes [14]. Erlotinib has FDA/EMEA approval for locally advanced or metastatic NSCLC as well as advanced or metastatic pancreatic carcinoma, and has also been studied in HNSCC. The response rate to EGFR-targeted therapies is smaller than expected, due to primary resistance [15] and to the development of acquired resistance [16]. In recurrent/metastatic HNSCC, monoclonal antibody therapies in clinical trials have demonstrated superior increases in OS and PFS than tyrosine kinase inhibitors [17, 18]. Therefore, it is important to gain knowledge of the molecular mechanisms of drug resistance since the identification of the tumors that rely on EGFR signaling for their growth is critical for the optimal selection of patients for therapy.

One molecule that has been shown to be involved in resistance to EGFR inhibitors in different tumor types is MET, the receptor tyrosine-kinase for hepatocyte growth factor (HGF) [19]. MET can activate many of the same downstream signaling pathways as EGFR, such as ERK1/2 and PI3K/AKT; additionally, it promotes tumor growth by affecting proliferation, anti-apoptosis, invasion, and angiogenesis in several tumor types [20]. In HNSCC, MET is expressed on epithelial cells and is activated by HGF through a paracrine mechanism. HGF is synthesized by stromal fibroblasts as an inactive precursor, and requires activation to generate responses via MET stimulation in the target cells [21]. Reports of lung, colorectal carcinoma, and glioblastoma have shown that MET is activated in the presence of EGFR blockade as a compensatory pathway, resulting in a mechanism of acquired resistance to EGFR inhibitors [22, 23]. MET and HGF are both consistently overexpressed in HNSCC [24], and such overexpression correlates with an aggressive disease and poor prognosis [25, 26]. Following several reports that established the HGF/MET pathway as an important driving force in HNSCC metastasis, and after some studies correlated its expression with the clinicopathological parameters and the survival of HNSCC patients [27], several clinical trials (http://clinicaltrials.gov) were conducted with HGF antagonists (rilotumumab, ficlatuzumab) and MET inhibitors (foretinib, crizotinib) in order to determine whether the inhibition of the HGF/MET pathway may be of therapeutic benefit in HNSCC patients [28].

In addition to HGF and MET overexpression, this pathway can also be activated through genetic alterations such as *MET*-activating mutations that, although rare in all tumor types, are certainly contributing to carcinogenesis. Two somatic activating *MET* mutations have been identified in HNSCC (Y1248C, and Y1253D), which increase the kinase activity of MET and subsequently lead to tumor proliferation and metastasis [29]. Additionally, evidence suggests that EBV and HPV infections are risk factors for the development of HNSCC. Viral infection has a prognostic impact on HNSCC, and of these, HPV-positive cancers have a more favorable prognosis [30], whereas the HPV-negative group, overwhelmingly made up of tobacco-related cancers, is the highest-risk group and has the worse prognosis [31]. However, few studies have investigated the association of the HGF/MET pathway expression/activation with HPV status [32].

Owing to the above mentioned, MET has been established as a marker of biological significance in cancer. We have investigated the impact on cetuximab sensitivity of HGF and MET overexpression, MET activation, *MET* gene status, and *MET* mutations in recurrent/metastatic HNSCC patients. We show that MET and p-MET overexpression are associated with poor outcome in recurrent/metastatic patients. In addition, we find that phosphorylation of MET is an independent prognostic factor in these patients. Taken together, our results support the idea that HGF/MET pathway might act as a resistance mechanism against EGFR inhibition in advanced HNSCC [33]. Consequently, a dual blocking strategy with anti-HGF/MET and -EGFR therapy may be an effective approach that would eventually benefit HNSCC patients who are resistant to other therapies.

## Methods

### Patients and tumor samples

A single-institution retrospective analysis including 57 consecutive HNSCC patients from Fundacion Jimenez Diaz Biobank (Madrid) was carried out, including clinical follow-up. The study examined 33 recurrent/metastatic patient samples (test group) along with 24 non-recurrent/metastatic patient samples (control group). Recurrent/metastatic patients were subsequently treated with cetuximab. Tissue microarrays were constructed with biopsy 1.0 mm cores from formalin-fixed and paraffin-embedded (FFPE) tumor biopsies obtained before treatment, using a semiautomatic tissue arrayer (Beecher Instruments, USA); they contained three cores per sample from representative areas of tumor.

### Protein abundance determination by immunohistochemistry (IHC)

For each case, FFPE samples were assayed for EGFR, HGF, total and phosphorylated MET using the following antibodies: EGFR (D38B1) rabbit mAb (Cell Signaling, USA), HGF (4C12.1) mouse mAb (Millipore, USA), MET (SP44) mouse mAb (Ventana Medical Systems, USA), and p-MET Y1234/1235 (3D7) rabbit mAb (Cell Signaling). Immunostaining was performed as described previously [34]. As a positive control, sections of NSCLC tumors with known marker expression were stained. Sections from the same specimens incubated with normal mouse and rabbit IgG2 instead of primary antibodies were used as negative controls. Antigen preservation in tissues was confirmed by assaying sections from the same tissue array for expression of phospho-tyrosines, using an anti-phosphotyrosine mAb (4G10, Millipore).

Stainings were evaluated by two pathologists (F.R. and E.G.). HGF was evaluated in tumoral stroma; EGFR, MET and p-MET were quantified in the membrane of tumor cells. In addition, a semiquantitative histoscore (Hscore) was calculated by estimation of the percentage of tumor cells positively stained with low, medium, or high staining intensity after applying a weighting factor to each estimate. The formula used was Hscore = (low %) × 1 + (medium %) × 2 + (high %) × 3, and results ranged from 0 to 300.

### HPV in situ hybridization

The Ventana Benchmark XT platform for ISH (Ventana) was used for HPV detection. Briefly, sections were assayed for *HPV* DNA by in situ hybridization with INFORM HPV-III Family-16 Probe(B) cocktail for 12 high-risk genotypes, and visualized using the ISH iVIEW PlusDetection Kit (Ventana). The high-risk HPV ISH test was scored as positive if there was any blue reaction product that co-localized with the nuclei of malignant cells.

The *digene* HC2 High-Risk HPV DNA Test (Qiagen, Germany) was used as a confirmatory assay for HPV detection. The test allows for the qualitative detection of 13 high-risk genotypes. Assays were performed following the manufacturer’s instructions and the chemiluminescent signals were measured in a DML instrument. Samples with processed values ≥1.0 are considered positives.

### Gene expression analysis by quantitative PCR

The levels of *EGFR* and *HGF* gene expression were determined using a quantitative RT-RealTime PCR assay on 5 × 10 μm sections of the FFPE biopsies, using an *ATP5E* gene as a housekeeping reference. Primers were designed to detect all variants according to the mRNA sequences NM_005228.3 for *EGFR*; NM_000601.4 (variant 1), NM_001010931.1 (variant 2), NM_001010932.1 (variant 3), NM_001010933.1 (variant 4), and NM_001010934.1 (variant 5) for *HGF*; and NM_006886.2, and NM_001001977.1 for *ATP5E*. qPCRs were conducted in a LightCycler480 II system (Roche Applied Science, Switzerland) using the following sets of primers: *EGFR*, 5′-GCTTGGATCCAAAGGTCATC and 5′-CAAGTGGATGGCATTGGAATC; *HGF*, 5′-GTGACCAAACTCCTGCCAG and 5′-CTTCTTTTCCTTTGTCCCTCTG; *ATP5E*, 5′-CCGGCGTCTTGGCGATTC and 5′-GATCTGGGAGTATCGGATG.

Relative *EGFR* and *HGF* expression ratios were calculated using the Pfaffl method [35] relative to the calibrator sample (MVP Human Breast Total RNA, Agilent Technologies, USA). The efficiencies of every primer pair were estimated by a standard curve (Additional file 1: Figure S1A).

### Dual-color in situ hybridization

The *MET* gene copy number was assessed by silver-enhanced in situ hybridization (SISH) on tissue microarray sections. Automated dc-SISH INFORM probes (Ventana) were performed on the Ventana Benchmark XT staining platform by labeling the 7q31 region that contains the *MET* gene and the centromeric alpha-satellite region, specific for chromosome 7, according to the manufacturer’s protocol. The following data were recorded for each sample: mean *MET* gene and mean *CEP7* copy number per cell and *MET*/*CEP7* ratio in 50 nuclei for each core. Evaluable results—at least one core with valid *MET* and *CEP7* counts—were obtained for all cases. The status of the EGFR gene was assessed by fluorescence in situ hybridization (FISH) using the LSI EGFR (7p12) FISH probe (Ventana), labeling the centromeric alpha-satellite region specific for chromosome 7 (spectrum green), and the EGFR gene region (spectrum orange), as recommended. The assessment of gene copy number was performed independently and blinded from IHC by two investigators (F.R. and S.Z.).

### Mutation analysis

Pyrosequencing was used to evaluate the status of selected Y1248C and Y1253D *MET* gene mutations on 4 × 10 μm sections from each tumor. Since both mutations localize very closely in exon 19, we used the same set of 3 primers. The sequences were as follows: *MET*.exon19, 5′-TGTCCTTTCTGTAGGCTGGATG and 5′-[Btn]AATACATTACCACATCTGACTTG; sequencing primer, 5′-GCTGATTTTGGTCTTGCC. Fifty ng of DNA were PCR amplified, modified, and finally pyrosequenced on a PyroMark ID system (Qiagen) following the manufacturer’s instructions. Cut-off value vas set to 8 % nucleotide mutation.

### Statistical analysis

Statistical analysis was carried out with IBM SPSS Statistics version 21.0. Overexpression criteria were defined by receiver operating characteristic (ROC) curve for each protein. ROC analysis was used to determine the optimal cut-off value based on the progression endpoint for each protein, in agreement with the methodology used in prognostic studies [36]. Amplification was defined by ≥3 copies in at least 2 of the 3 tumor areas studied. To analyze correlations between HGF, MET, and p-MET protein expression and clinical-pathological variables, we used the χ^2^ test (Fisher’s exact test) or Mann–Whitney test. Overall survival (OS) was defined as the time elapsed from the date of initial diagnosis to the date of death from any cause or the date of last follow-up. Progression-free survival (PFS) was defined as the time from treatment to either progressive disease or death from any cause, censored at last contact [11]. Survivals were analyzed by the Kaplan–Meier method (median follow-up 75 months) and curves were compared using the log-rank test. Multivariate analysis, including continuous quantitative and qualitative clinical-pathologic parameters, was carried out using the Cox proportional hazards model. All statistical tests were conducted at the two-sided 0.05 level of significance. This work was performed in accordance with the Reporting Recommendations for Tumor Marker Prognostic Studies (REMARK) guidelines [37].

## Results

### MET expression and activation in recurrent/metastatic HNSCC

**Fig. 1:**
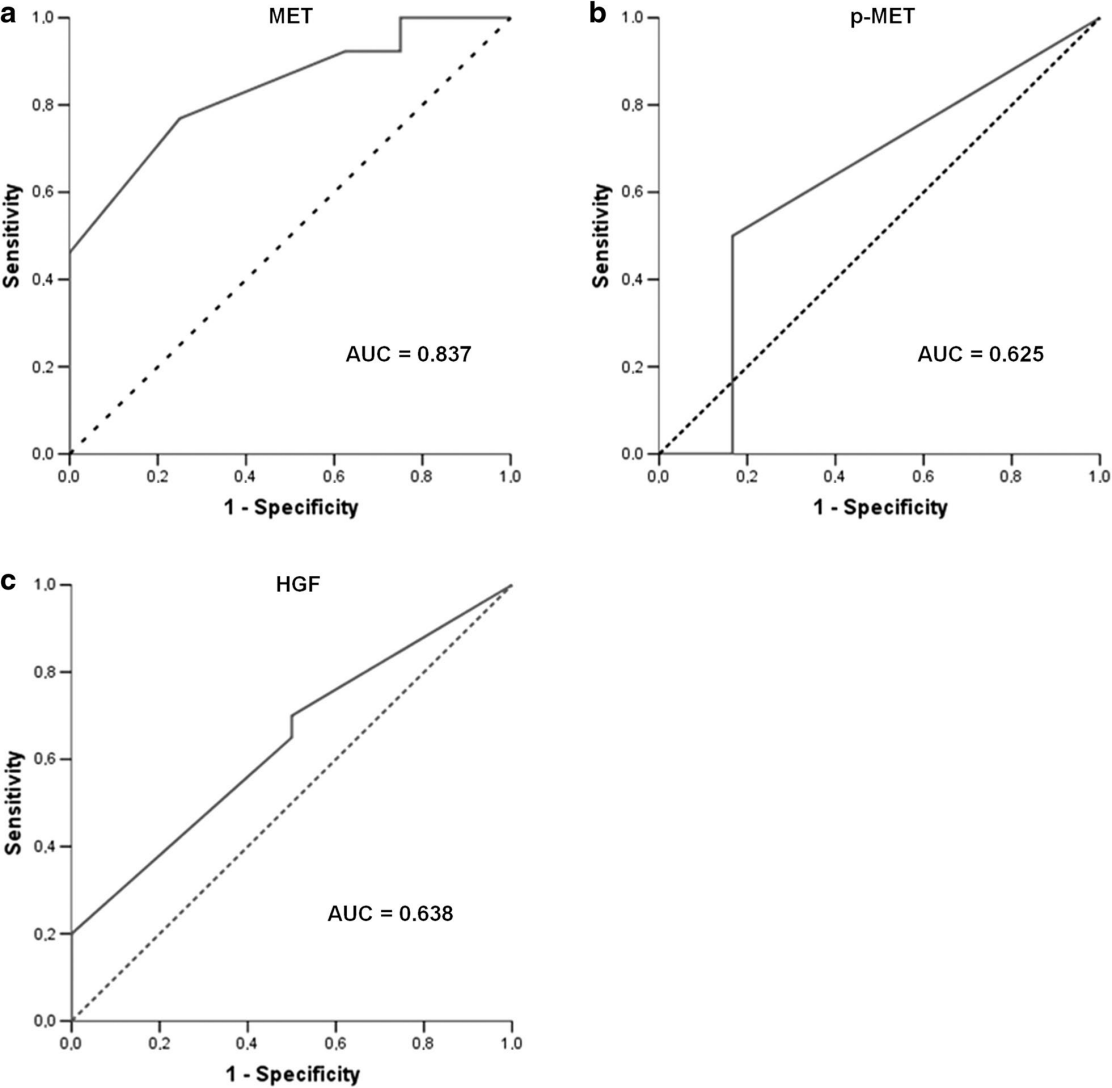
MET (**a**), p-MET (**b**), and HGF (**c**) overexpression thresholds in the cohort of HNSCC patients. ROC curves were used to calculate the optimal biomarker thresholds based on the progression endpoint for each protein. These scores, in correspondence, defined protein overabundance. *Full lines* represent the ROC curves; in *dot lines*, the diagonal reference lines

Clinical-pathological features for both test and control groups of patients are summarized in Table 1. HPV status was negative for the majority of the samples (1 positive case by both determination methods). We defined the optimal overexpression threshold that could be used as prognostic marker for MET, p-MET, and HGF. The area under the ROC curve (C-statistic) was calculated for every case based on the progression endpoint for each protein (Fig. 1). MET achieved an area under the curve (AUC) of 0.837, whereas p-MET and HGF had lower and comparable diagnostic performance (AUC ~0.630 in both cases). The optimal cut-off points for MET, p-MET, and HGF were calculated at Hscores of 120, 10, and 100, respectively.

**Table 1:** Clinical characteristics of test and control groups of patients

	Test group				Control group				*P* value
	n	%			n	%			
Age [mean (range)]	61 (38–80)				64 (41–80)				1
Sex									0.059
Male	31	96.2			18	75.0			
Female	2	3.8			6	25.0			
Performance status									0.444
0	1	3.0			0	0			
1	32	97.0			19	79.2			
ND					5	20.8			
Smoking history									0.912
Current smoker	11	33.3			9	37.5			
Former smoker	21	63.6			14	58.3			
Never smoker	1	3.0			1	4.2			
Primary site									0.952
Oral cavity	7	21.2			6	25.0			
Oropharynx	7	21.2			4	16.7			
Hypopharynx	6	18.2			4	16.7			
Larynx	12	36.4			10	41.7			
ND	1	3.0							
Failure sites									
Locoregional	28	87.5			NA				
Distance	19	59.4			NA				
Both	10	31.3			NA				
Therapeutic regimen			Length^a^	Follow-up^b^			Length^a^	Follow-up^b^	
Cetuximab	15	45.5	4–64 (12)	15–76 (30)	NA				
Cetuximab/platinum/5FU	13	39.4	4–56 (22)	6–74 (33)	NA				
Cetuximab/taxane	5	15.1	20–32 (24)	24–26 (25)	NA				
Chemotherapy (CDDP)	NA				5	20.8	3 (3)	9–121 (40)	
No chemotherapy					13	54.2			
ND					6	25			
Skin toxicity									
Rash grade 1	7	21.2			NA				
Rash grade 2	11	33.3			NA				
Rash grade 3	5	15.2			NA				
ND	10	30.3							
Hypomagnesemia									
Yes	6	18.2							
No	15	45.5							
ND	12	36.4			24				

*NA* not applicable, *ND* no data available

^a^Cycles, range (median)

^b^Months, range (median)

**Fig. 2:**
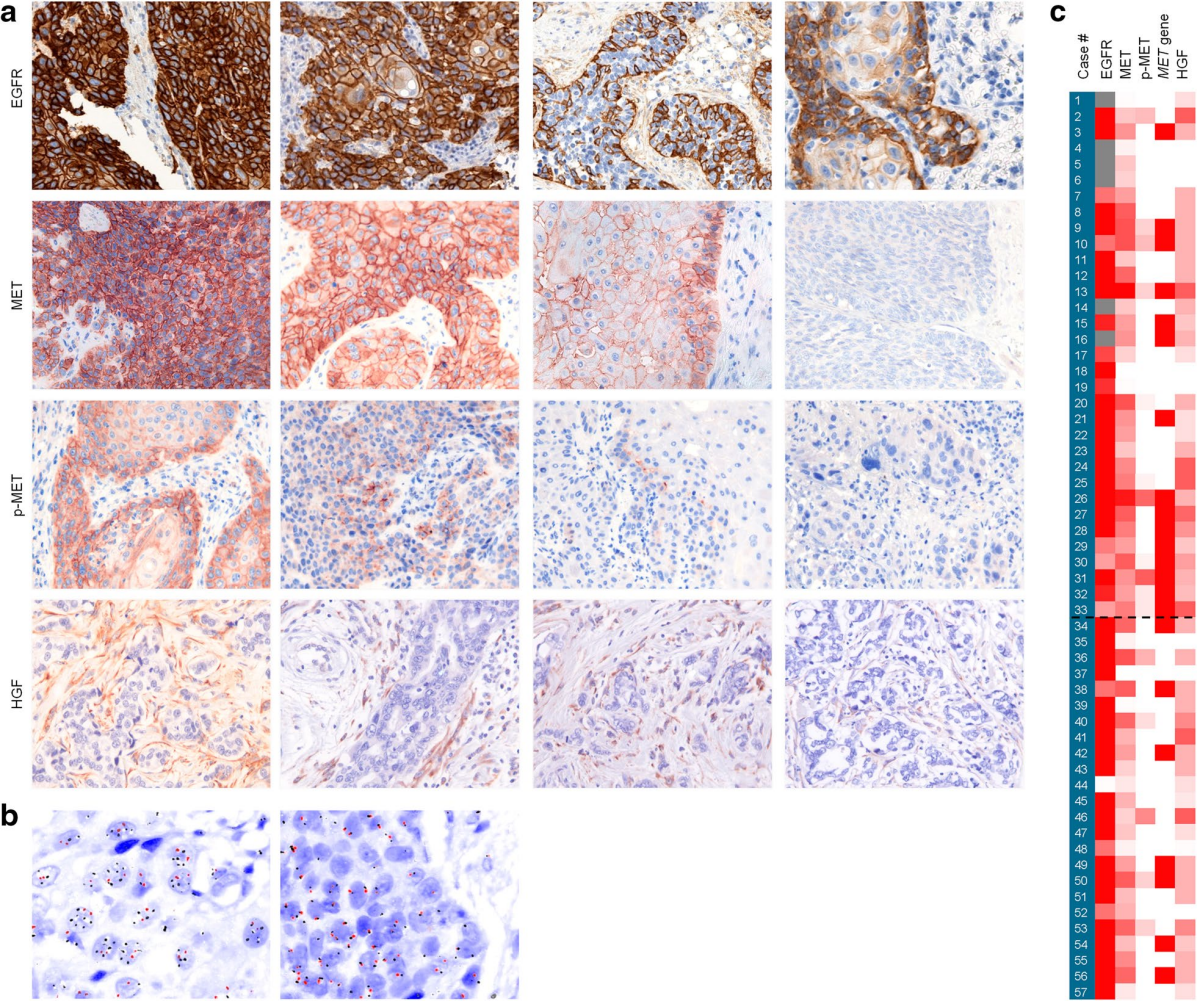
HGF/MET pathway expression and activation in recurrent/metastatic HNSCC. **a** Representative pictures showing a range of EGFR, MET, p-MET, and HGF expression levels observed by IHC in human HNSCC. **b** *MET* identification in FFPE tissue samples by SISH. *Left panel* detail of a *MET*-amplified case (clusters of *black dots* are seen in the nuclei of tumoral cells, representing several copies of the *MET* locus; as opposed to just two *red dots* per cell, corresponding to the centromeric region on chromosome 7). *Right panel* non-amplified sample. **c** Protein expression levels and gene amplification in the complete series. Case numbers are ordered from test (#1–33) to controls (#34–57), and a *dashed line* has been drawn in between the two groups. The *color* intensity of the *boxes* is indicative of abundance level in each column, either protein level by IHC (EGRF, MET, p-MET, HGF) or gene amplification level by SISH (*MET* gene). Expression levels are indicated in a color gradient, from *white* (minimum) to *red* (maximum), with missing data in *gray*

EGFR elevated expression was confirmed in all the cases, both at the gene (Additional file 1: Figure S1B) and protein level (Fig. 2a). MET expression, on the other hand, revealed a heterogeneous pattern in the tumors studied (Fig. 2a). Expression ranged from homogeneous intense staining to complete absence of signal (range 10–300; median 120). Differences in intensity of MET expression were occasionally detected in the same tumor. In total, overexpression of MET was detected in 33 (58 %) of all patients: in 22 (67 %) of the test samples and in 11 (46 %) of the control samples (Table 2; Fig. 2c). The detection of phosphorylated Y1234/1235 MET also showed a grading along the series (Fig. 2a) (range 0–180; median 80). p-MET overexpression was detected in 17 (30 %) patients in the whole series, in 12 (36 %) of the test, and in 5 (21 %) of the control cases (Table 2).

**Table 2:** EGFR, MET, p-MET, HGF protein expression levels and MET gene amplification in the complete series

Expression levels	test Group		Control group		*P* value
	n	%	n	%	
EGFR					0.053
Low	0	0.0	2	8.3	
Medium	2	6.1	3	12.5	
High	25	75.7	19	79.2	
ND	6	18.2			
MET					0.066
Low	8	24.2	16	66.7	
Medium	16	48.5	5	20.8	
High	9	27.3	3	12.5	
p-MET					0.060
Low	27	81.8	20	83.3	
Medium	4	12.1	4	16.7	
High	2	6.1	0	0.0	
MET gene					0.092
Yes	17	51.5	7	29.2	
No	16	48.5	17	70.8	
HGF					0.369
Low	9	27.3	11	45.8	
Medium	17	51.5	10	41.7	
High	7	21.2	3	12.5	

Different ranges included those cases with low (0–33 %), medium (34–66 %), or high (67–100 %) IHC expression levels determined as a percentage of the Hscore

*MET* gene amplification was next assessed for the 57 cases (Fig. 2b). Twenty-two cases (39 %) exhibited amplification of the region corresponding to the *MET* locus, 15 of them corresponding to test samples (Table 2). Although the fraction of overexpressing/amplified cases was quantitatively higher in the test group, the differences for these markers between the two groups were not found to be statistically significant. In consequence, we calculated the correlations between markers for the complete series. Significant correlations were found between *MET* gene amplification and overexpression (Table 3) (*P* = 0.004). In our series, all the cases displaying gene amplification except one also showed high levels of receptor expression, confirming the straightforward link between genomic dose and protein synthesis. All the test cases that showed *MET* amplification were also overexpressing the protein (100 %). In the case of the control samples, 6 out of the 7 (86 %) patients with amplification showed high levels of MET expression. Additionally, other significant associations were found between receptor activation with gene amplification (*P* = 0.047) and receptor expression (*P* = 0.013).

**Table 3:** Correlations between biomarkers expressed as *P* values (Chi square test)

	MET overexpression	p-MET overexpression	HGF overexpression	*MET* amplification
MET overexpression		0.013	0.517	0.004
p-MET overexpression	0.013		0.001	0.047
HGF overexpression	0.517	0.001		0.786
*MET* amplification	0.004	0.047	0.786	

Expression levels as determined by IHC. Results include all 57 patients

### *HGF* gene is moderately overexpressed in HNSCC

Elevated levels of ligand HGF are coupled with activation of the MET receptor. The expression levels of the *HGF* gene in the samples were verified by qPCR (Additional file 1: Figure S1B). The number of positive cases was 33 (58 %), 18 (55 %) of whom from the test group and 15 (62 %) from the control group. There was a reasonable agreement with the HGF protein determination by IHC. Additionally, a similar pattern of sample heterogeneity was visualized in the immunohistochemical expression of the HGF protein. The signal for HGF was mainly visualized in the surrounding stroma but not in the tumoral cells, as opposed to the images of MET and p-MET (Fig. 2a). With respect to the potential role of *HGF* gene overexpression in MET activation, 8/17 samples (47 %) that had shown p-MET overexpression did in fact hold elevated mRNA levels of its ligand. Importantly, HGF overexpression was associated with MET phosphorylation (*P* = 0.001), suggesting a paracrine activation of the receptor (Table 3).

### *MET* mutation analysis

Sequencing screening was performed for the two most frequent Y1248 and Y1253 *MET* mutations in HNSCC. One case (2 %) was deemed positive with 12 % mutation TGT in position Y1248. In addition to the pyrosequencing analysis, de novo sequencing was performed in the amplified 34-nucleotide length region spanning the Y1248 and Y1253 area in the *MET* locus, in order to check for the presence of hotspots surrounding the 2 targeted codons 1248 and 1253.

### Prognostic role of the MET pathway in cetuximab-treated HNSCC patients

**Fig. 3:**
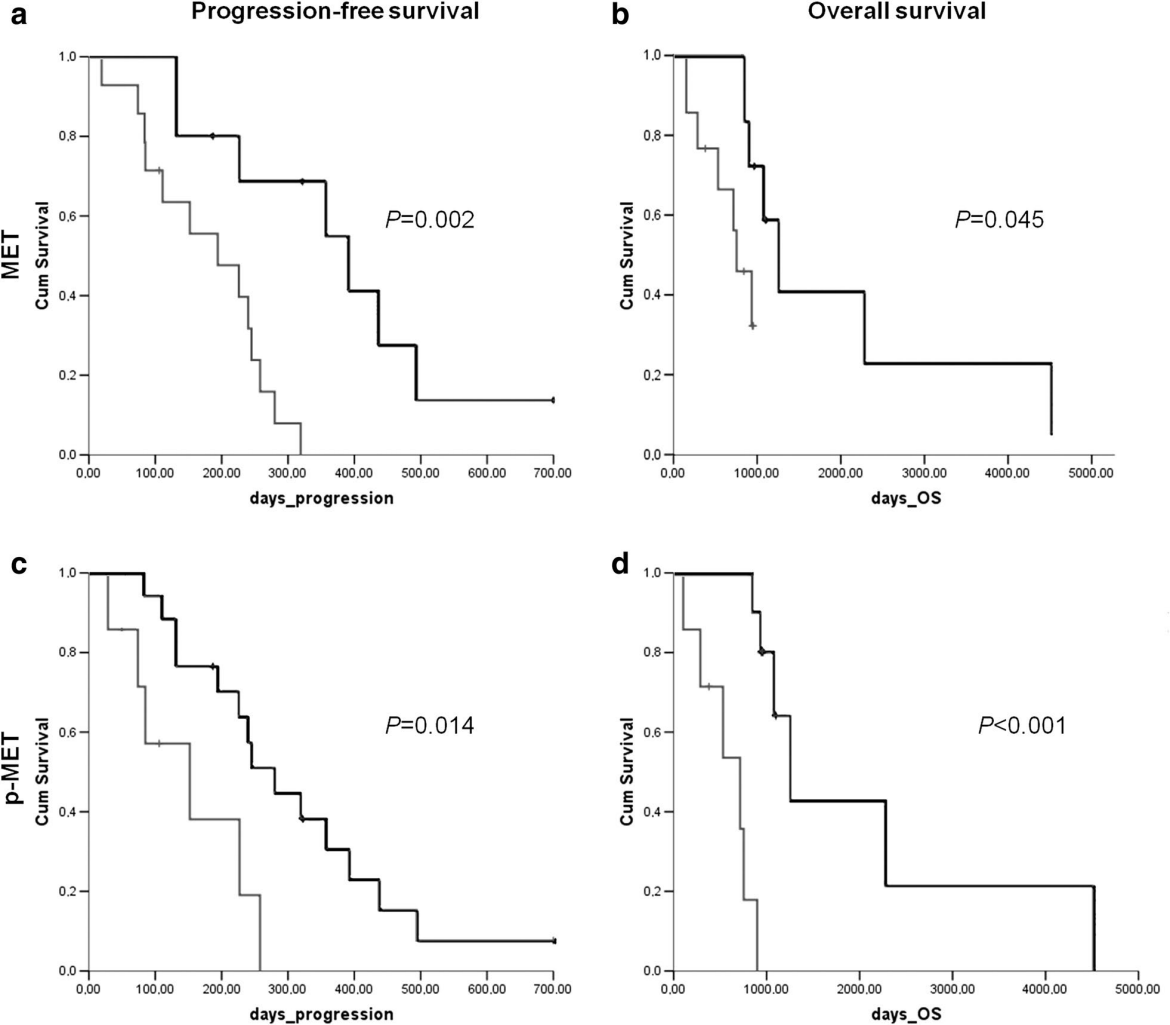
Prognostic role of MET and p-MET in cetuximab-treated HNSCC patients. **a** Progression-free survival (PFS) for MET. **b** Overall survival (OS) for MET. **c** PFS for p-MET. **d** OS for p-MET. In *light gray line*, patients with MET overexpression; in *black line*, patients without MET overexpression

To provide data regarding the prognostic impact of MET expression and activation in human HNSCC under a cetuximab-based treatment, we performed a survival analysis of our series of patients, stratifying the status of the markers. Kaplan–Meier curves for MET and p-MET in PFS and OS were calculated. Both MET and p-MET overexpression revealed a poor outcome in HNSCC patients from the test group (Fig. 3). Log-rank testing showed a significantly worse outcome in MET-overexpressing patients for PFS (*P* = 0.002) and OS (*P* = 0.045). p-MET expression was also significantly associated with a poor clinical outcome for OS (*P* < 0.001). Patients with p-MET overexpression had worse prognosis (median PFS 15 months; median OS 18 months) compared with p-MET negative/low expression cases (median PFS 37 months; median OS 48 months). Moreover, p-MET overexpression also correlated with worse PFS (*P* = 0.014). Multivariate Cox analysis in the test group (Table 4) confirmed the independent prognostic significance of p-MET for PFS (HR 6.5; 95 % CI 1.5–8.9) and for OS (HR 8.2; 95 % CI 0.2–14.6). No significant association of HGF overexpression with clinic-pathological parameters was detected. Histological staging did not show any significant impact in the survival of the patients.

**Table 4:** Multivariate cox regression models for progression-free survival (PFS) and overall survival (OS) in the 33 cetuximab-treated patients

	PFS			OS		
	HR	95 % CI	*P* value	HR	95 % CI	*P* value
Smoking history			0.268			0.786
Ex-smoker	1.0			ND		
Smoker	2.2	0.2–20.9		ND		
Non-smoker	0.4	0.2–1.9		ND		
Primary site			0.184			0.389
Larynx	1.0			1.0		
Oropharynx	0.3	0.5–2.2		0.3	0.1–4.6	
Oral cavity	2.8	0.4–10.2		2.1	0.4–6.3	
Hypopharynx	2.1	0.3–8.3		2.6	0.1–10.2	
Histological grade			0.090			0.719
Well-differentiated	1.0			1.0		
Moderately differentiated	4.6	2.5–7.6		0.9	0.1–3.2	
Poorly differentiated	3.2	2.3–8.5		1.9	0.1–4.1	
Alcohol	2.1	0.7–10.3	0.381	6.2	0.1–34.2	0.413
MET overexpression	7.6	4.6–10.4	0.060	4.9	0.1–8.5	0.070
p-MET overexpression	6.5	1.5–8.9	0.002	8.2	0.2–14.6	0.022
HGF overexpression	6.6	1.2–8.4	0.059	2.2	0.2–2.1	0.110

Firstly, univariate analysis was performed for the descriptive variables, and then we executed a multivariate analysis on those significant variables. Since MET, p-MET and HGF expression are associated with each other, we performed separate analysis for each marker

*HR* hazard ratio, *CI* confidence interval, *ND* not enough data available

## Discussion

We have retrospectively addressed the correlation of HGF/MET pathway overexpression and activation with cetuximab response in samples from patients that later developed recurrent/metastatic HNSCC, and we have concluded that it correlated with worse outcome in patients treated with a cetuximab-based regimen. The point that samples were collected prior to treatment suggests that it may act as a primary resistance mechanism for EGFR inhibitors. Previous studies have already reported that primary resistance can decrease the response rate to EGFR-targeted therapies [15]. An additional activation of the HGF/MET pathway—that may function as a compensatory route—has been reported in some other tumors [19, 38], but the first description of MET overexpression in HNSCC patients [39] was published only recently. In agreement with previous studies, our work demonstrates that MET was expressed in 58 % of HNSCC patients in our total series, and that p-MET was expressed to a lesser extent, in just 30 % of cases, with no significant differences between the test and control groups. Our series also shows a tight correlation between *MET* gene amplification and MET overexpression in HNSCC patients (Fig. 2c). These findings reveal a direct link between gene amplification, gene high expression, and protein overabundance. Given that several studies indicate that MET activation is responsible for approximately 20 % of resistance to EGFR inhibitors [29, 40], our results suggest a condition of potential primary resistance.

Biomarker levels were determined in samples collected at the time of initial diagnosis but not at the time of recurrence/metastasis diagnosis, in order to avoid levels alterations due to additional chemotherapy treatments. Although expression levels might fluctuate from diagnosis to recurrence/metastasis, biomarkers at the time of diagnosis are characteristic of every individual, and their profiles possess prognostic value to evaluate the progression of the disease.

Resistance to EGFR-inhibition therapies is a growing concern in HNSCC clinical practice, due to both primary resistance and to the development of acquired resistance by many patients that only respond transiently to therapy with EGFR-targeted drugs [41]. The study of resistance to cetuximab therapy in HNSCC closely echoes the strategies used to uncover the mechanisms of resistance to tyrosine kinase inhibitors (gefitinib, erlotinib) in other tumor types [17, 42]. Setting aside the mutations on the EGFR kinase domain (cetuximab targets the extracellular domain of EGFR, and therefore its mechanism of action does not affect the tyrosine kinase domain), *MET* amplification represents the most obvious focus of research, in terms of prevalence. EGFR shares important downstream signaling targets with MET, another transmembrane tyrosine-kinase receptor, including ERK1/2, PI3K/AKT, STAT3, and PLCγ. The HGF/MET signaling pathway can be activated by *MET* genomic amplification [19], by overexpression of the ligand HGF [38] or the MET receptor kinase, or by its activating mutations [29, 43]. In all cases, MET activation occurs by phosphorylation of any of several residues in the tyrosine-kinase domain. A sustained activation of common EGFR and MET downstream targets leads to malignant growth [44].

In vitro studies of MET and EGFR have shown that a single amplified receptor tyrosine-kinase can determine growth and survival of different cancer cell lines (lung, gastric). It was found that the amplified MET was constitutively activated, suggesting an oncogene addiction phenomenon that was required for cell survival [45]. Our present results would now demonstrate that this in vitro requirement may correlate with in vivo growth in patients.

Furthermore, our data suggest that, in case of gene amplification, HGF liberation may not be required for MET activation. Conversely, it may be necessary for those cases with no *MET* amplification. In fact, HGF overexpression did not correlate with *MET* amplification (*P* = 0.786), although it was associated with MET phosphorylation (*P* = 0.001), suggesting a paracrine activation of the receptor (Table 3). It seems that, in these cases, a greater concentration of HGF and/or proximal interaction of tumor and stromal cells are critical for the activation of the MET pathway (although HGF measurements include the stromal pool, and thus they do not necessarily equate with its active form). As has been demonstrated in murine models, while HGF was secreted by HNSCC tumor-derived fibroblasts, but not by HNSCC cells, MET was expressed and functional in HNSCC cells [39]. Addition of HGF induced MET phosphorylation, leading to the activation of AKT and ERK, and tumor proliferation, confirming that HGF acts mainly as a paracrine factor in HNSCC cells.

Regarding the third possible mechanism of MET activation, two somatic constitutively active MET mutations have been identified in lymph node metastases of HNSCC (Y1248C and Y1253D) [46]. Only one Y1248C mutation (2 %) was found in the *MET* locus (none in Y1253), probably due to the small probability of finding low-prevalence mutations in our small series. Although these mutations are barely detectable in primary tumors from patients, it has been shown that cells carrying the mutations are selected during the metastatic spread [46]. This is in agreement with our hypothesis that MET pathway activity mechanisms preexist in the HNSCC population, and subsequent treatment with anti-EGFR therapy may expose processes of resistance. Intriguingly, we found four hotspots near these two positions, wherein the proportions of non-canonical nucleotide incorporation were significantly elevated. This point merits further research, as it might indicate that these areas in the *MET* gene tend to accumulate mutations in the tumoral cells, and it might be related to the mechanisms and consequences of the previously described Y1248 and Y1253 mutations in HNSCC.

From a clinical perspective, the most relevant finding of this study was the prognostic role of MET and p-MET expression in HNSCC. In the literature debate surrounds the possible prognostic role of total MET in human cancer, with many studies suggesting a negative prognostic role [20, 47], while others indicate the contrary [40, 48], and some studies even discern no relationship at all. With respect to MET phosphorylation, our data suggest that it may an independent prognostic factor in these patients. Since this is a surrogate marker of receptor activation, our finding is consistent with an adverse role of activated MET receptor in HNSCC, supporting the findings of previous reports that correlate increased MET activation with resistance to cetuximab in both HNSCC cell lines [19] and patient-derived xenografts [49]. In other tumor types similar conclusions about the role of activated MET have been drawn [34].

Since the first reports of EGFR-targeted therapy in HNSCC, it has been known that EGFR expression is needed for cetuximab response, although EGFR expression does not predict response. Our data suggest that combined treatments of a MET inhibitor and cetuximab may be cumulative, and therefore dual blocking of EGFR and HGF/MET pathways could be a reasonable therapeutic option for clinical practice. Among the various options for inhibiting MET, most efforts concern to the use of small molecule inhibitors of MET, or antibody inhibitors of MET or its ligand, HGF [50].

## Conclusions

Due to the limited number of patients in our study and the fact that our analysis did not include a validation cohort, we must assume the conclusions as a preliminary indication of the role of the HGF/MET pathway regarding cetuximab resistance in HNSCC. We have confirmed that the pathway is overexpressed and overactivated in HNSCC patients. This activation of MET is constitutive in those patients with *MET* gene amplification, while HGF overexpression is required for MET activation in non-amplified cases. In recurrent/metastatic HNSCC patients, MET and p-MET overexpression are associated with poor outcome, and phosphorylation of MET is considered an independent prognostic factor in these patients. Finally, and in accordance with previous suggestions in different cancer types, we propose that the HGF/MET pathway might act as a primary resistance mechanism for EGFR inhibitors. The absence of a correlation between HGF/MET pathway activity and outcome in the control group is a significant finding that reinforces this hypothesis. Consequently, we would contemplate a dual blocking of both routes, in a combination therapy of EGFR and HGF/MET tyrosine-kinase inhibitors, for those patients with recurrent or metastatic HNSCC.

## Additional file

Additional file 1:
**Figure S1.** Representative qRT-PCR analysis for EGFR and HGF mRNA copy levels. **A.** Standard curves for target EGFR, HGF and reference probe ATP5E, as determined by 5 or 8 triplicate points over a range from 0.05 to 10 ng. The efficiencies (E) were calculated from the slope of the standard curves according to the equation: E = (10^[-1/slope]^-1), by using 5 or 8 dilution points. The efficiencies were determined as follows: E_EGFR_= 1.974; E_HGF_= 2.091; E_ATP5E_= 1.902. RFU, relative fluorescence units. **B.** qPCR amplification curves of EGFR, HGF and ATP5E probes, in triplicate, for a representative sample, showing the distance with the calibrator sample.
