# Supplementary figures and images for: Activation of MET pathway predicts poor outcome to cetuximab in patients with recurrent or metastatic head and neck cancer
Source: J Transl Med. 2015 Aug 29;13:282. doi: 10.1186/s12967-015-0633-7 (PMC4552997; doi:10.1186/s12967-015-0633-7)

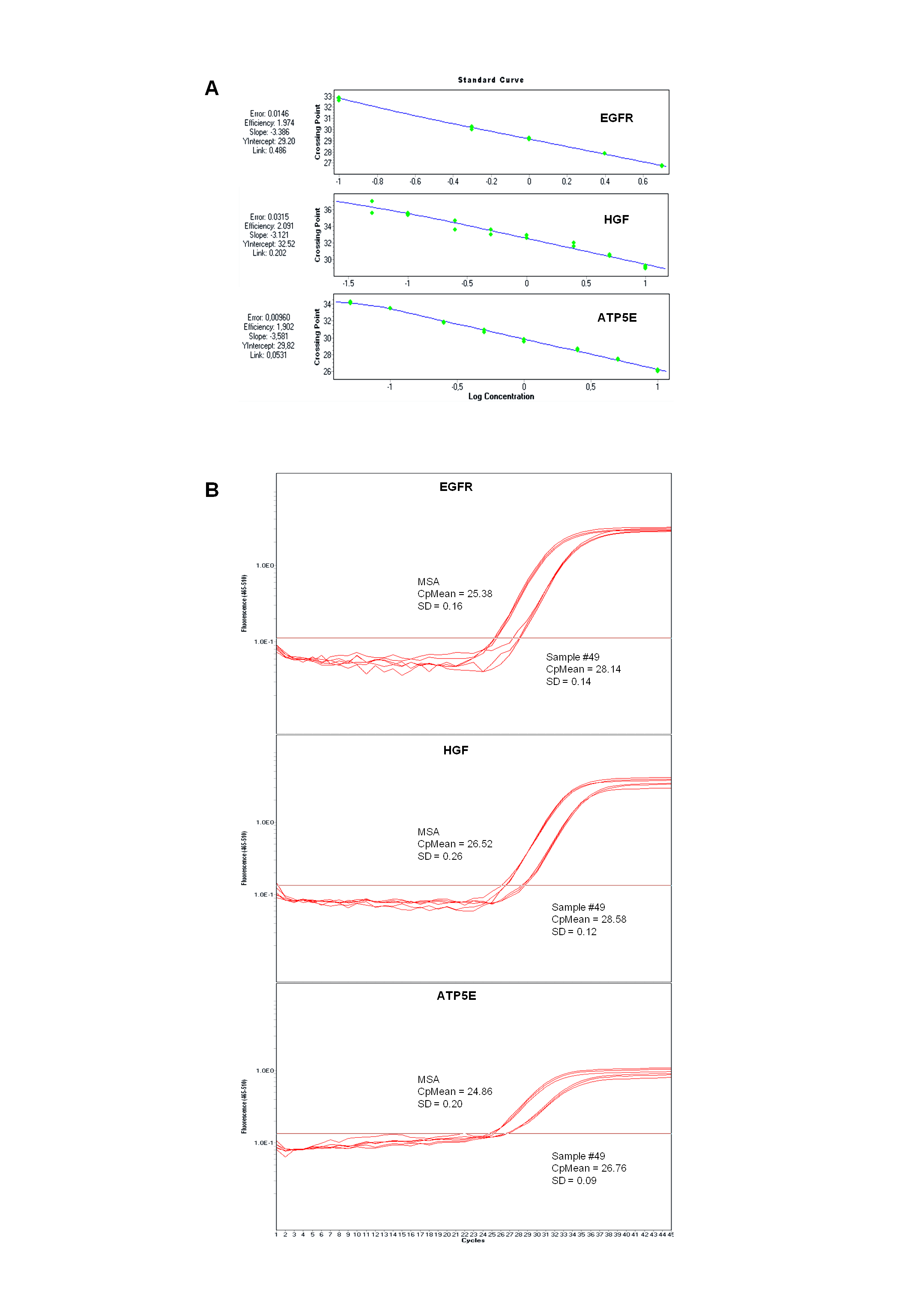

Supplement: Additional file 1: — Figure S1. Representative qRT-PCR analysis for EGFR and HGF mRNA copy levels. A. Standard curves for target EGFR, HGF and reference probe ATP5E, as determined by 5 or 8 triplicate points over a range from 0.05 to 10 ng. The efficiencies (E) were calculated from the slope of the standard curves according to the equation: E = (10[-1/slope]-1), by using 5 or 8 dilution points. The efficiencies were determined as follows: EEGFR= 1.974; EHGF= 2.091; EATP5E= 1.902. RFU, relative fluorescence units. B. qPCR amplification curves of EGFR, HGF and ATP5E probes, in triplicate, for a representative sample, showing the distance with the calibrator sample. [file 12967_2015_633_MOESM1_ESM.tiff]
